# Defining new radiological patterns to improve classification of Bosniak III and IV cystic renal masses

**DOI:** 10.1007/s00261-025-05092-7

**Published:** 2025-07-02

**Authors:** Carmen Sebastià, Lledó Cabedo, Sergio Jiménez-Serrano, Héctor Alfambra, Daniel Corominas, Marc Comas-Cufi, Josep Puig, Carlos Nicolau

**Affiliations:** 1 Hospital Clínic de Barcelona, Barcelona, Spain; 2 August Pi i Sunyer Biomedical Research Institute, Barcelona, Spain; 3 University of Girona, Girona, Spain

**Keywords:** Bosniak cyst, Computed tomography, Malignancy, Follow-up, Renal cell carcinoma

## Abstract

**Objectives:**

We aimed to compare Bosniak III and IV cystic renal masses (CRM) using Bosniak classification version 2005 (BC-v2005) versus BC-v2019 and analyze radiological findings and patterns of benignity and malignancy.

**Methods:**

We retrospectively reviewed all Bosniak III-IV CRMs using BC-v2005 at our center with four-phase CT confirmed pathology, between January 2014 and June 2019. Two radiologists independently re-evaluated each lesion using both BCs, including findings of benignity and malignancy. Bosniak III-IV CRMs were classified in four radiological patterns: inflammatory (CRM-IP), cystic nephroma (CRM-CNP), papillary (CRM-PP), and clear cell (CRM-CCP). Characteristics of patterns were compared.

**Results:**

Out of 97 patients, 57 Bosniak III-IV CRMs met the inclusion criteria. Twenty-four (42.10%) lesions were reclassified as solid tumours using BC-v2019. Any lesion was downgraded to IIF or lower. Nine (15.78%) lesions were upgraded to Bosniak III-IV CRMs by BC-v2019, despite benign lesions. The presence of acute-angle nodules was the only statistically significant sign of malignancy (*p* < 0.01). All benign lesions, not considered solid, were included in the two patterns of benignity, characterized by thickened septa and/or wall and without acute-angle nodules. All malignant CRMs presented two patterns of malignancy, proposed as acute-angle nodules. All clear cell carcinomas presented with hyperenhancing acute-angle nodules in the arterial phase. Pattern classification was better for BC-v2005 and BC-v2029, differentiating benign and malignant CRM.

**Conclusion:**

The proposed four radiological patterns improved performance than BC-v2019 in classifying benign lesions (CRM-IP and CRM-CNP) and malignant ones (CRM-PP and CRM-CCP), with wall and septa thickening and acute-angle nodules as relevant biomarkers.

**Supplementary Information:**

The online version contains supplementary material available at 10.1007/s00261-025-05092-7.

## Introduction

Bosniak Classification (BC) was conceived in 1986 and updated in 2005 (BC-v2005) and 2019 (BC-v2019) [1–3]. With the BC-v2005, Bosniak I and II CRM were classified as benign and did not require follow-up. Bosniak IIF CRM had low malignancy probability (4–16%) requiring at least five years of imaging follow-up. Bosniak III–IV CRM have a high probability of malignancy (50% and 90%, respectively), indicating surgery [2]. This led to resection of many benign lesions (50% Bosniak III and 10% Bosniak IV cysts) [2]. BC-v2019 aimed to improve inter-reader agreement and emphasize specificity over sensitivity, avoiding overdiagnosis and overtreatment [3].

Recent literature shows BC-v2019 downgrades benign CRM previously classified as Bosniak III by BC-v2005, reducing unnecessary resections [4–6]. A recent meta-analysis found 79% of BC-v2019 Bosniak III CRM were malignant [6]. However, malignancy in BC-v2019 class IIF CRM was high (46%) [7]. Studies suggest a risk of downgrading malignant lesions to categories not requiring follow-up [4–7]. Despite improvements, BC-v2019 still has weaknesses.

CRM, even histologically malignant ones, are usually indolent, making active surveillance (AS) reasonable [8]. Management recommendations for Bosniak IIF, III, and IV CRM currently include AS [9]. Studies report long survival rates in patients with Bosniak III and IV CRM undergoing AS [9]. Follow-up can use CT, MRI, or contrast-enhanced ultrasound (CEUS) [10].

This study aims to compare Bosniak III and IV CRM classified by BC-v2005 versus BC-v2019 and analyze radiological findings and patterns of benignity and malignancy to avoid unnecessary surgery of benign lesions in BC-v2019 Bosniak III–IV CRM.

## Materials and methods

We retrospectively reviewed all CRM classified as Bosniak III and IV in our center using the BC-v2005 and treated with partial or radical nephrectomy or with AS with previous biopsy, between January 2014 and June 2019. Inclusion criteria were: 4-phase CT protocol performed, lesion classified as BC-v2005 III/IV CRM, and histopathological diagnostic confirmation. CRMs that did not meet these characteristics of study protocol, classification or histopathological confirmation were excluded.

This retrospective study was approved by the Research Ethics Committee. Informed consent was not required as this was a retrospective study based on previously performed CT and pathology reports, which did not influence patient management.

The CT protocol included unenhanced and enhanced phases (corticomedullary, nephrographic, and excretory phases) with 5 mm and 2 mm reconstruction sections in the axial and coronal planes. This was the standard protocol for renal mass characterization and presurgical mapping performed at our center.

Two radiologists (SJ and CS) with 3- and 20-years’ experience independently re-evaluated CRM using BC-v2005 and BC-v2019 and assigned the resulting classification to each cyst. Discrepancies were resolved by a third abdominal radiologist with 21 years of experience (CN). Patients’ medical records were reviewed and the following clinical data were collected: sex, age, American Society of Anesthesiologists score, tobacco use, arterial hypertension, body mass index, serum creatinine (Cr), glomerular filtration rate (GFR) before surgery, tumor size, previous renal surgery, definitive treatment, ischemia time, pathology results, serum Cr and GFR 1 month after surgery, recurrence, and treatment of recurrence.

The classification of these cysts with BC-v2005 was compared with BC-v2019 criteria by CT and correlated with pathological findings. Radiological findings evaluated included: >25% enhancing tissue based on visual inspection, presence and number of septa, presence of wall, enhancement of septa and wall, septa and wall maximum thickness, irregularity of septa and wall, presence of nodules with obtuse and acute angles and maximum diameter of nodules and enhancement, using HU Hounsfield Units (HU), of cystic and solid CRM areas before and after contrast injection (arterial phase). CRM solid areas were defined as areas with a change of > 20 HU compared to unenhanced and enhanced CT or two different enhanced CT phases. Nodules were considered hyperenhancing if they enhanced at least as much as renal cortical parenchyma in the arterial phase and hypoenhancing if less.

**Fig. 1:**
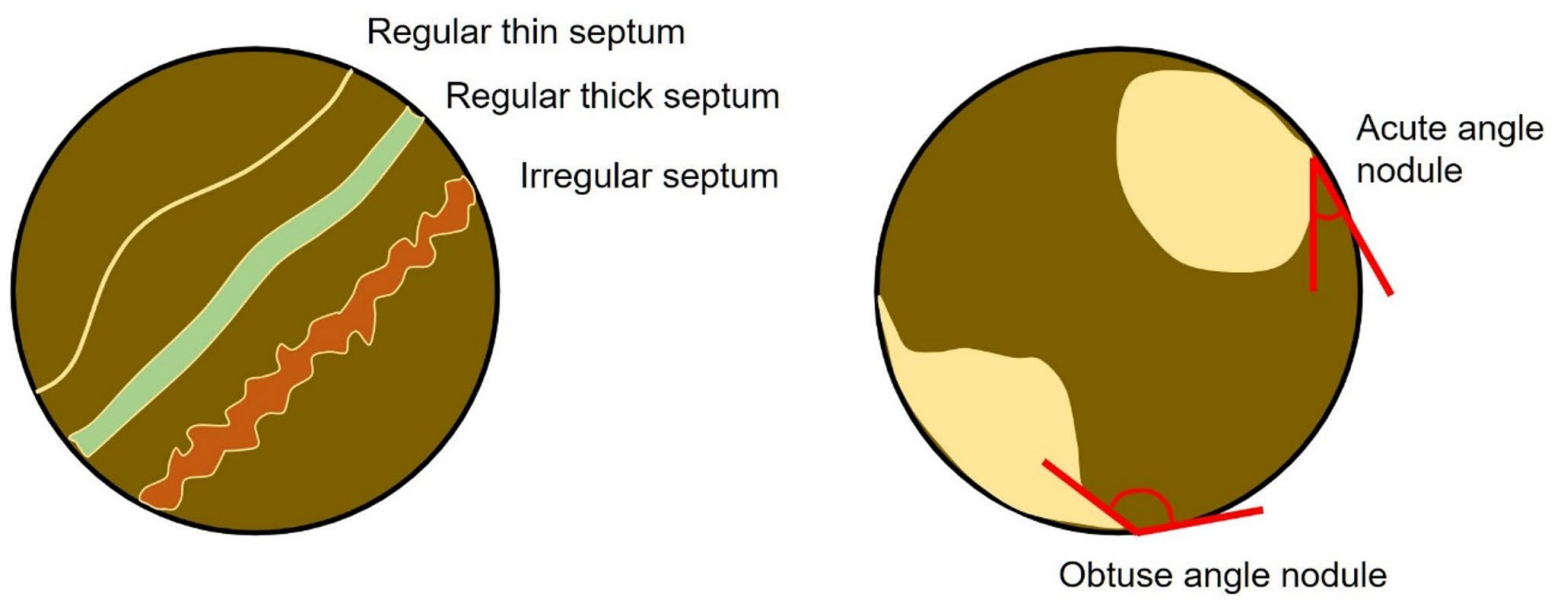
Visual definition of different types of septa and nodules

We defined thin septa as regular septa less than 3 mm in thickness, thick septa as regular septa with 3 or more mm in thickness. A septum or wall is considered irregular if its margins are not smooth, regardless of its thickness. Nodules with acute angles are those that present an acute angle in their contact with the wall or septum and are considered obtuse angle nodules if this angle is obtuse (Fig. 1).

**Fig. 2:**
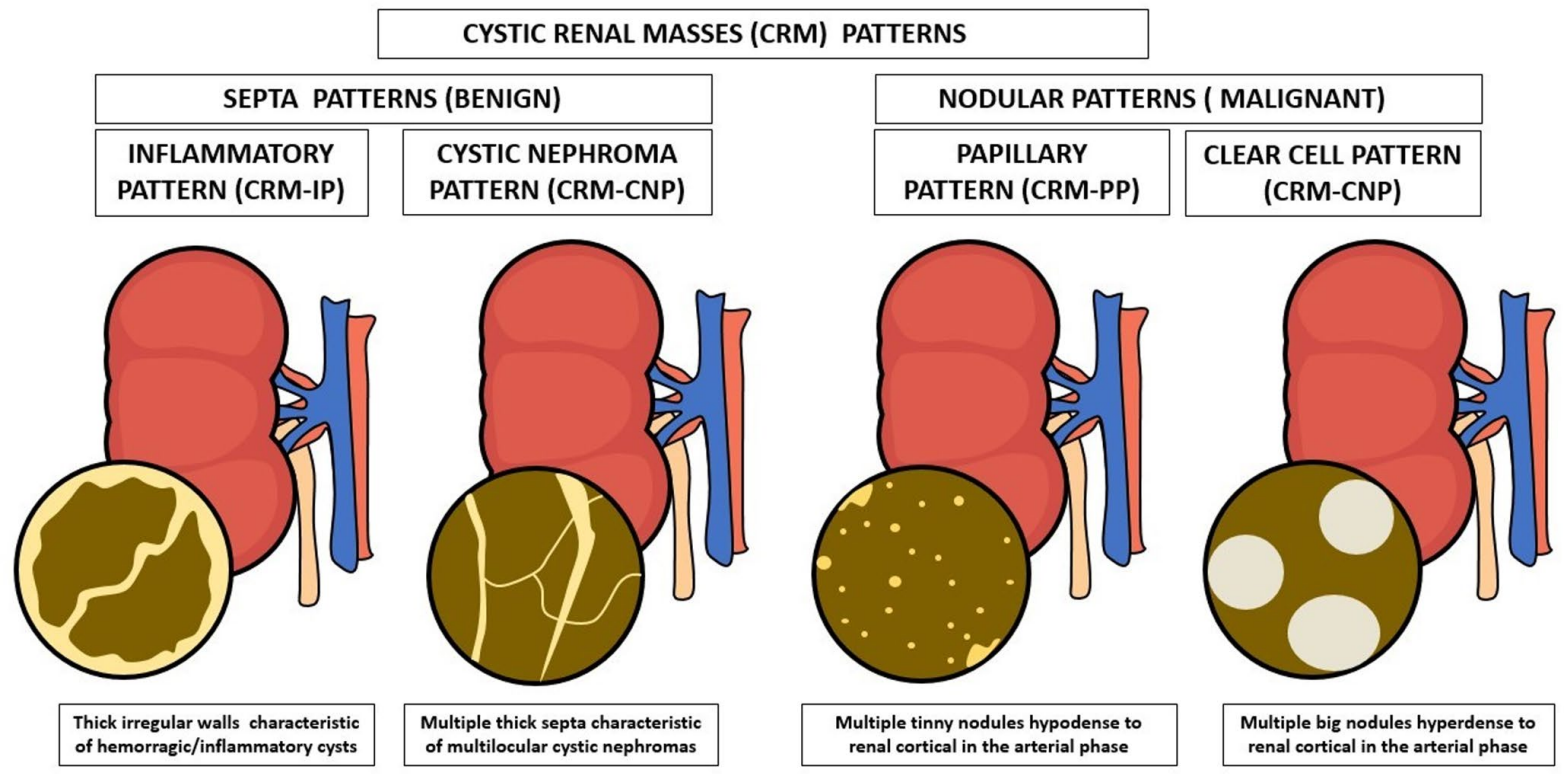
Radiological CRM patterns

We described 4 CRM radiological patterns based on experience and literature (Fig. 2).

Two radiological patterns were identified corresponding to benign CRM, we called them Septa Patterns:

1. CRM Inflammatory Pattern (CRM-IP). CRM with enhancing irregular mural wall with or without enhancing irregular septa, presenting focal mural thickening of ≥ 3 mm This pattern is characteristic of hemorrhagic and residual inflammatory cysts [11].
2. CRM Cystic Nephroma Pattern (CRM-CNP). CRM with multiple enhancing septa, with or without thickened enhancing walls, and focal septa thickening ≥ 3 mm. This pattern is characteristic of multilocular cystic nephroma (MCN) and is mostly found in young patients without other renal cysts [12].
  Two clearly differentiated patterns were observed in the malignant CRM group called Nodular Patterns.
3. CRM Papillary Pattern (CRM-PP). CRM with heterogeneous hypodense content in all phases and with small enhancing nodules. These nodules were hypodense in the arterial phase compared to renal parenchyma. This pattern is typical of papillary renal cell carcinomas (pRCC) [13].
4. CRM Clear Cell Pattern (CRM-CCP). CRM presented with large enhancing nodules hyperdense in arterial phase compared to renal parenchyma. This pattern appears specifically in Clear Cell Renal Cell Carcinomas (ccRCC) [14].

Each CRM was included in the most similar pattern by two radiologists (SJ and CS), with a third for discrepancy cases (CN). Radiological characteristics of patterns were analyzed to identify significant differences. Detailed characteristics of these four patterns are also presented.

### Statistical analysis

Demographic and imaging data were compared between the groups. Descriptive analysis included frequencies and percentages for categorical variables and medians and interquartile range (IQR) for continues variables. Fisher’s exact tests and Kruskal-Wallis rank sum tests were used to contrast variables. ROC analyses were performed to discriminate variables according benign and malignant lesions. All statistical analyses were performed using R (version 3.6.1, R Foundation for Statistical Computing, Vienna, Austria, https://www.R-project.org).

## Results

A total of 56 patients with 57 CRM from 97 patients met the inclusion criteria. Forty CRM were excluded (27 for not having 4-phase CT protocol, 11 for not being considered BC-v2005 III and IV after revision, and 2 because pathology was insufficient). The characteristics of the cohort are summarized in Table 1.

Pathologists confirmed malignancy in 43 (75.4%) CRM (21/43 (48.8%) pRCC, 20/43 (46.5%) ccRCC, 1/43 (2.3%) chromophobe RCC, and 1/43 (2.3%) metastasis), being the most common histological finding of the whole sample (75.4%). Fourteen (24.6%) CRM were benign, with MCN (8/14 (57.1%)) being the most common histological finding. Pathological results are shown in Table 1.

**Table 1:** Sample characteristics of Bosniak III and IV cystic renal masses

Characteristic	Whole sample (*n* = 57 lesions)
Male sex, mean ± SD	38 (67.8)
Age [years], mean ± SD	60.1 ± 10.8
*Anatomopathological tumor type, n (%)*	
Papillary renal cell carcinoma	21
Clear cell renal cell carcinoma	20
Multilocular cystic nephroma	8
Inflammatory simple cyst	3
Oncocytoma	2
Hemangioma	1
Cromophobe renal cell carcinoma	1
Metastasis	1
Hypertension, n (%)	36 (64)
Body mass índex, mean ± SD	28.1 ± 5.8
Serum creatinine [mg/dl], mean ± SD	1.1 ± 0.6
Glomerular filtration rate [ml/min], mean ± SD	76.0 ± 18.9
Largest lesion diameter [cm], mean ± SD	3.2 ± 1.4
*Previous renal surgery, n (%)*	
Partial nephrectomy	2 (3.6)
Radical nephrectomy	2 (3.6)
Kidney transplant	4 (7.1)
*Definitive treatment, n (%)*	
Active surveillance (biopsy)	1 (1.75)
Partial nephrectomy	50 (87.7)
Radical nephrectomy	6 (10.5)
Ischemia time (min), mean ± SD	20.6 ± 7.1
Serum creatinine at 1 month after surgery, [mg/dl] mean ± SD	1.2 ± 0.6
Glomerular filtration rate at 1 month after surgery, [ml/min] mean ± SD	68.6 ± 20.6
Local recurrence, n (%)	1 (1.75)

Demographic characteristics of the 56 patients with Bosniak III and IV cystic renal masses. Results are expressed as mean ± standard deviation (SD) for continuous variables and as absolute number and percentage for categorical variables

*GFR* Glomerular filtration rate,*ASA* American Society of Anesthesiologists score,*BMI* body mass index

**Fig. 3:**
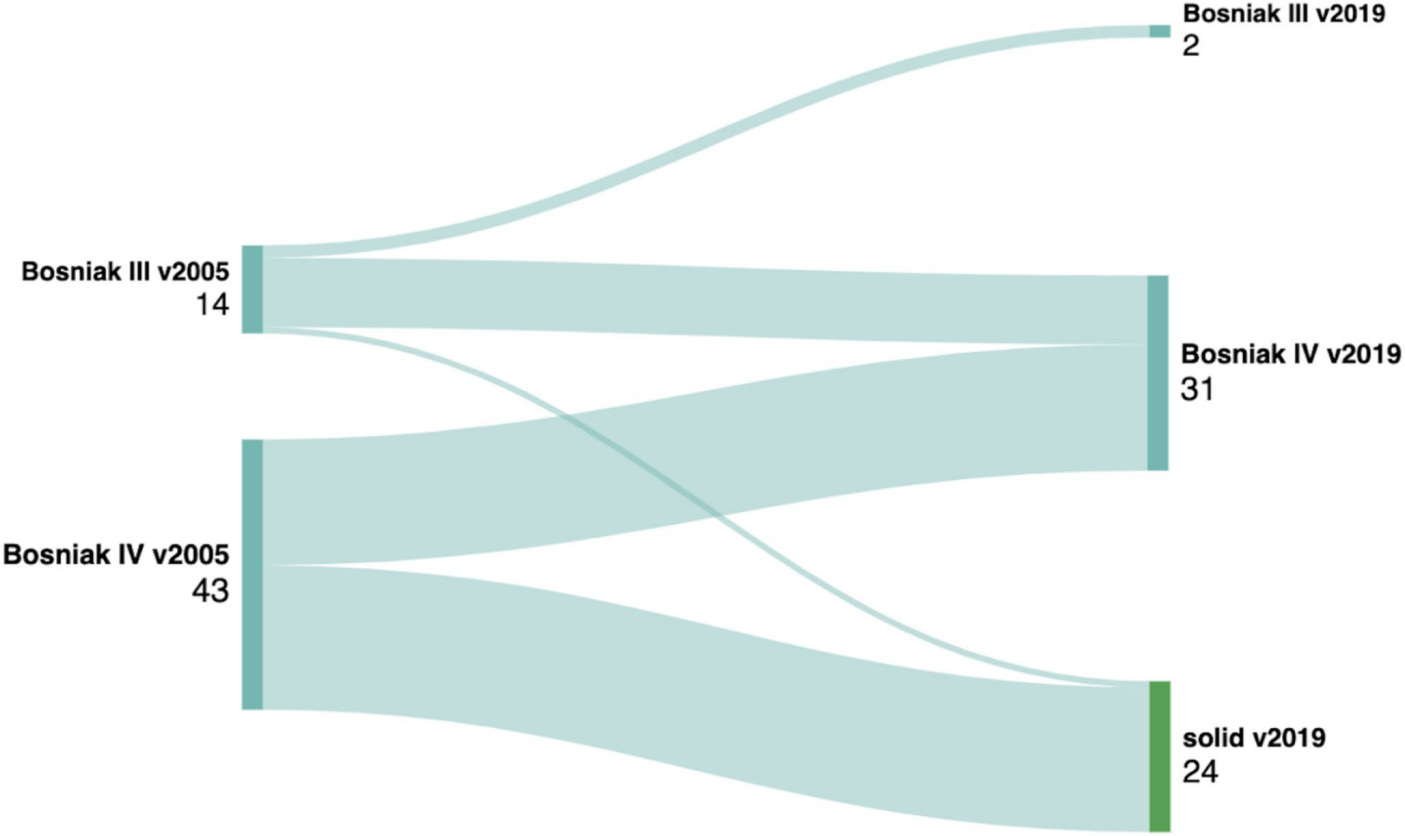
Discrepancy between cystic renal masses classification according to the Bosniak classification v2005 versus v2019

There were 14 (24.6%) BC-v2005 Bosniak III CRM and 43 (75.4%) BC-v2005 Bosniak IV CRM. We found significant discrepancies between BC-v2005 and BC-v2019 in classifying CRMs, as shown in Fig. 3. One of the most important observation was that 24 (42.10%) BC-v2005 Bosniak IV CRM were re-classified as BC-v2019 solid tumours due to > 25% of enhancing tissue. Of them, 21 (87.5%) were malignant and three (12.5%) were benign, corresponding to one haemangioma and two oncocytomas. Similarly, 12 of 14 (85.7%) BC-v2005 Bosniak III CRMs were upgraded BC-v2019 Bosniak IV. Of them, 9 were benign having irregular septa or walls with nodular thickening of > 3 mm.

However, there were lesions that did not change their classification; 2 of 14 (14.3%) BC-v2005 Bosniak III CRMs corresponded to BC-v2019 Bosniak III, and 17 of 43 (39.5%) BC-v2005 Bosniak IV were BC-v2019 Bosniak IV (Fig. 5). No BC-v2005 Bosniak III/IV CRM was downgraded to IIF or lower by BC-v2019.

Interreader agreement was moderate between the two readers for grading lesions by BC-v2019 (kappa = 0.56). Major disagreements were in considering enhancing thickened irregular septum or wall or obtuse-angle nodules, and the precise measurement of septa and wall thickness in millimeters.

Malignant lesions presented more solid component (44% vs. 21%; *p* < 0.001) and presence of acute angle nodules (95% vs. 0%; *p* < 0.001).

**Fig. 4:**
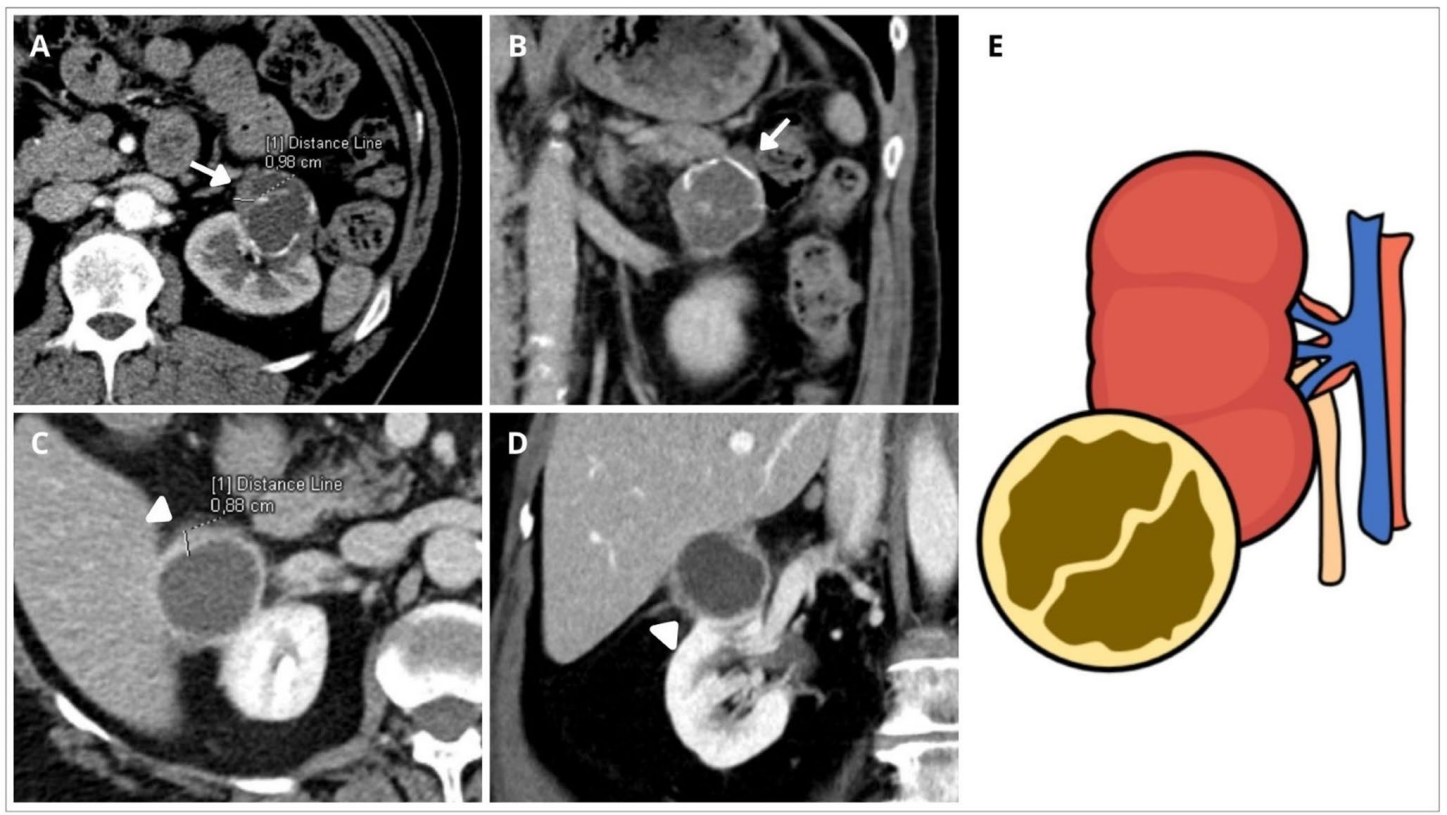
Cystic renal mass with Inflammatory Pattern (CRM-IP). **A**, **B** Axial and coronal views of enhanced CT of a CRM located in the upper pole of the left kidney with irregular wall and septa (arrows). Note nodular irregular wall thickness of 9,8 mm in size in **A** (arrow), and mural and septa calcifications. This case was considered Bosniak III by BC-v2005 and was update to Bosniak IV by BC-v2019. **C**, **D** Right upper pole cyst (arrowhead) with wall nodular irregular thickening 8,8 mm in size, measured in **C**. This cyst was also upgraded from BC-v2005 Bosniak III to Bosniak IV with BC-v2019. **E** shows a schema of the CRM-IP defined as a lesion with irregular mural wall without convex acute margin nodules. Inflammatory pattern always appear in benign CRM

**Fig. 5:**
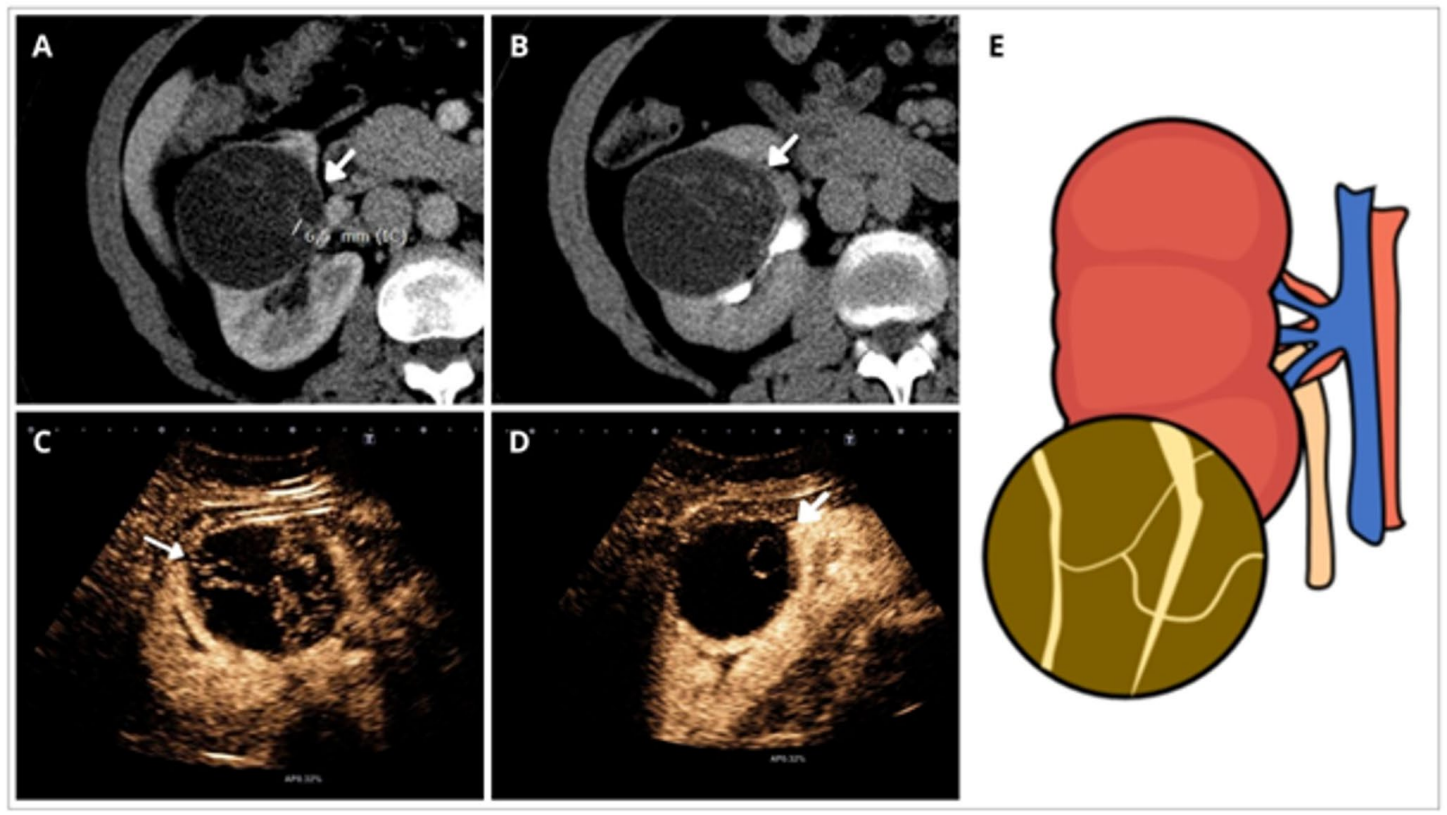
Cystic renal mass with Cystic Nephroma pattern (CRM-CNP). **A**, **B** Axial CT images of right CRM with multiple enhancing irregular septa in the nephrographic and excretory phases (arrows). One of the irregular septa measures 6,5 mm and is considered as Bosniak IV by BC-v2019. **C**, **D** CEUS of the same case demonstrate multiple enhancing thick irregular septa (arrows). **E** shows a schema of the CRM-CNP defined as a lesion with multiple septa and focal thickening without acute-angle nodules. Note that this pattern was always found in young patients without other renal cysts

**Fig. 6:**
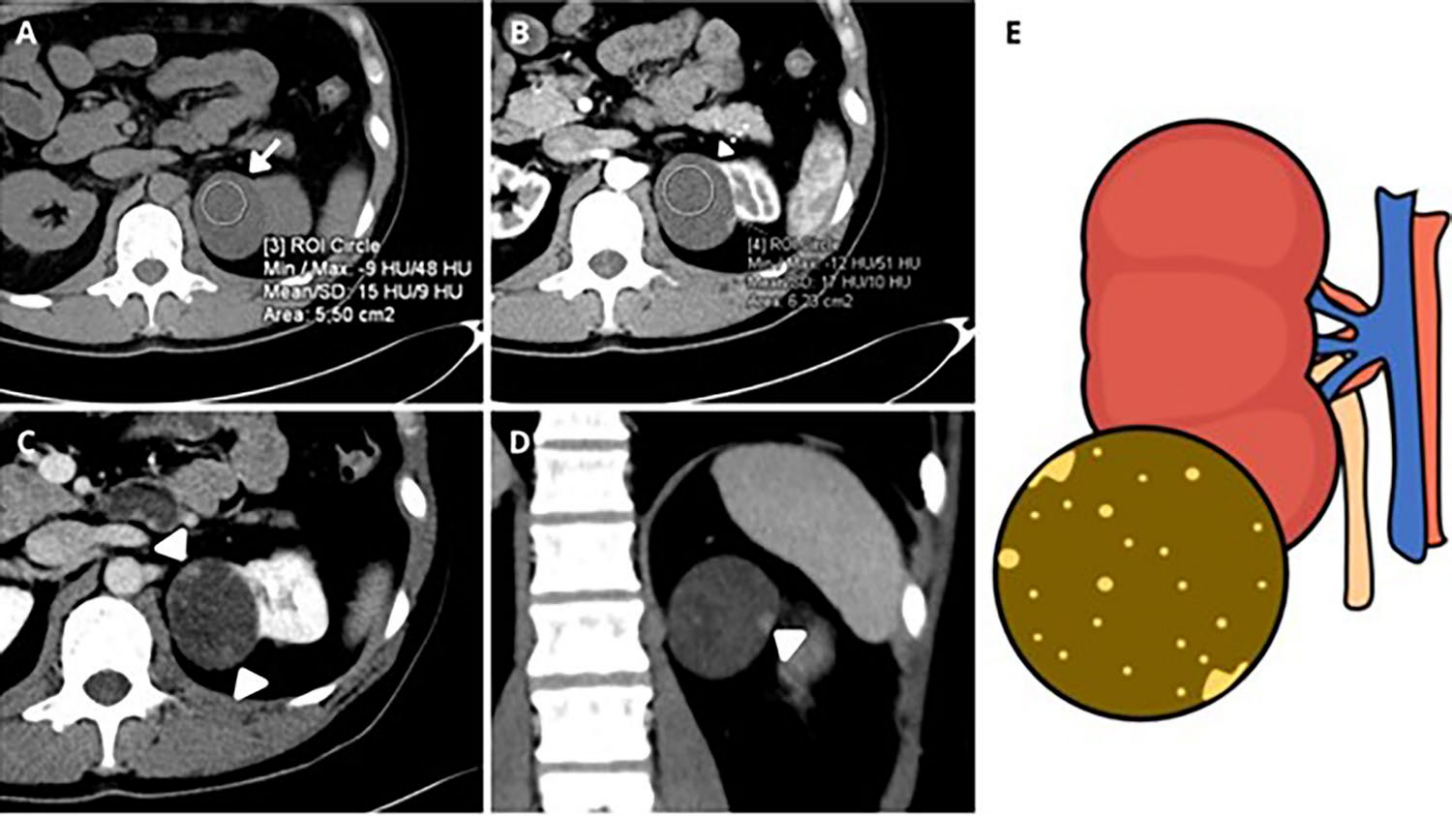
Cystic renal masses with Papillary Pattern (CRM-PP). **A**, **B** Axial unenhanced and arterial phase enhanced CT depict left CRM in the left kidney upper pole that was not enhanced after contrast administration (15 HU in unenhanced CT and 17 HU in arterial phase, less than 20 HU of change) (arrows). No nodules can be seen in the arterial phase. **C**, **D** Nephrographic phase in axial and coronal views. Multiple acute-angle nodules can be seen in the nephrographic phase that make up a mottled pattern with very small slightly enhanced nodules (arrowheads). Pathology confirmed papillary renal cell carcinoma. **E** shows a schema of the CRM-PP defined as a lesion with a heterogeneous hypodense acute-angle nodules that showed low enhancement in the arterial phase, indistinctly of the presence of nodules with obtuse-angle nodules

**Fig. 7:**
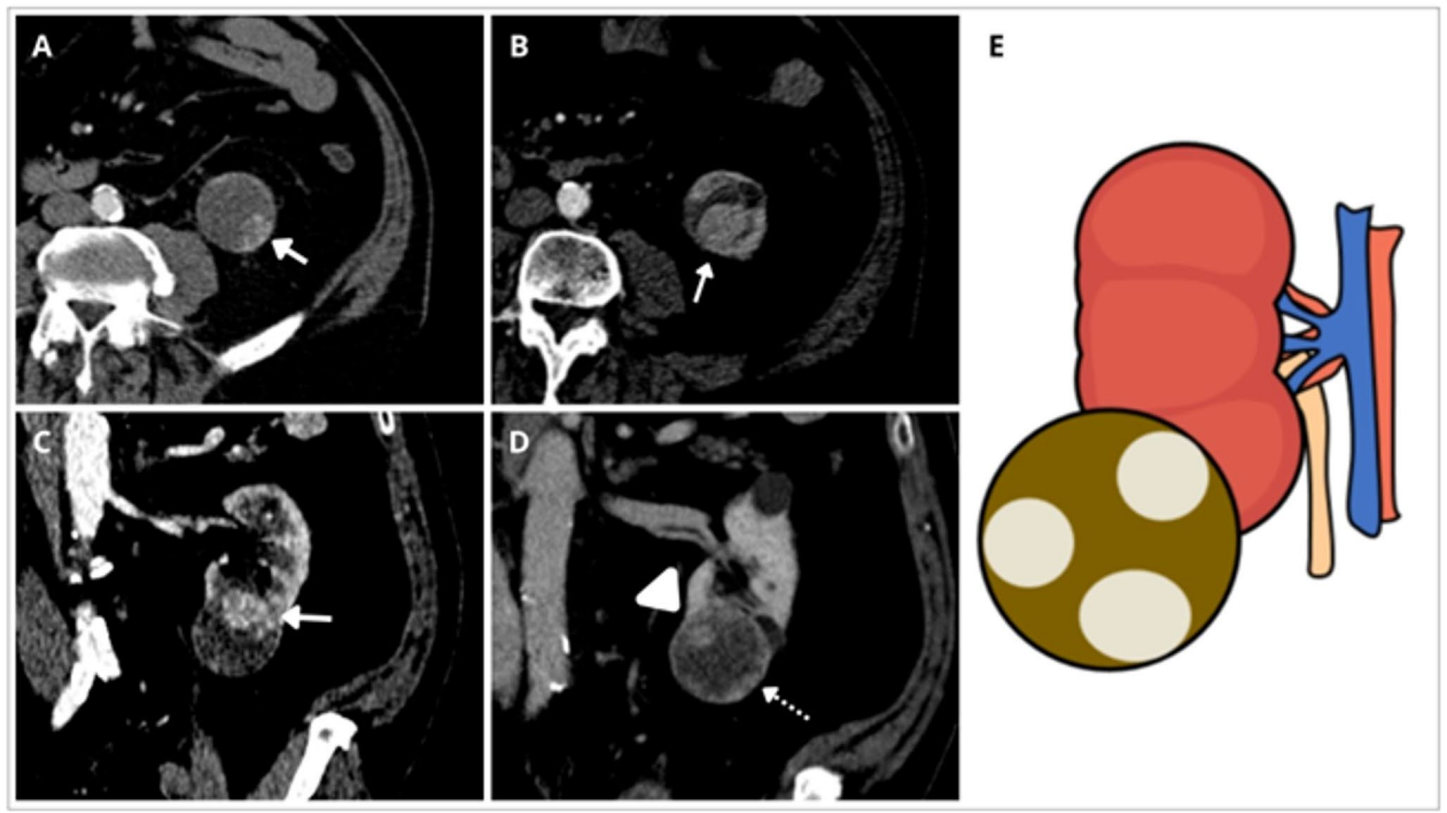
Cystic renal mass with Clear Cell Pattern (CRM-CCP). **A–C** Left lower pole cystic renal mass with hyper-enhancing mural acute-angle nodules in the arterial phase in axial and coronal views (arrows). **D** depicts enhanced mural nodules with acute (arrowhead) and obtuse (discontinuous arrow) angles in the coronal view of the nephrographic phase. Hyper-enhancement of mural nodules in the arterial phase that was found in the ccRCC in our series was the most characteristic finding that helps to differentiate these nodules from the hypodense nodules in the arterial phase that was observed in most pRCC in our series. **E** Shows a schema of the CRM-CCP defined as a lesion with nodules with predominantly convex acute margins hyperenhancing in the arterial phase

Radiological and pathological findings were analysed for the 4 patterns proposal to identify statistically significant differences (Table 2. All CRM-IP lesions had thick, irregular walls. All CRM-CNP lesions had no thick irregular walls but multiple focal thickened septa (≥ 3). All CRM-PP and CRM-CCP lesions presented acute-angle nodules; however, in CRM-PP group, nodules were smaller (5 mm (IQR, 0–8) vs. 13 mm (9–14); *p* < 0.001). CRM-CCP showed higher arterial phase attenuation of solid nodular component than CRM-PP (232 HU (225–240) vs. 90 HU (77–125); *p* < 0.001). Most ccRCC presented with CRM-CCP pattern (84.61%), and most patients with pRCC presented papillary pattern (88.88%) (Figs. 4, 5, 6 and 7) (Table 2).

**Table 2:** Radiological characteristics of CRM patterns

Variable	Inflammatory pattern (*n* = 3)	Cystic nephroma pattern (*n* = 8)	Papillary pattern (*n* = 9)	Clear cell pattern (*n* = 13)	*p*-value
Age [years]	69 (65, 73)	52 (47, 54)	65 (50, 71)	69 (61, 74)	0.03
*Histology, n (%)*					< 0.001
• Clear cell renal cell carcinoma	0 (0)	0 (0)	1 (11)	11 (77)	
• Papillary renal cell carcinoma	0 (0)	0 (0)	8 (89)	3 (23)	
• Multilocular cystic nephroma	0 (0)	8 (100)	0 (0)	0 (0)	
• Imflamatory Cyst	3 (100)	0 (0)	0 (0)	0 (0)	
*BC v2005, n (%)*					< 0.001
• III	3 (100)	8 (100)	1 (11)	1 (8)	
• IV	0 (0)	0 (0)	8 (89)	12 (92)	
BC v2019, n (%)					0.074
• III	0 (0)	2 (25)	0 (0)	0 (0)	
• IV	3 (100)	6 (75)	9 (82)	13 (100)	
*Type of lesion, n (%)*					< 0.001
• Benign	3 (100)	8 (100)	0 (0)	0 (0)	
• Malignant	0 (0)	0 (0)	9 (100)	13 (100)	
Wall present (%)	3 (100)	4 (50)	1 (11)	10 (77)	0.005
Wall maximum thickness, [mm]	5 (4.5, 5.5)	0.5 (0, 1.5)	0 (0, 0)	2 (1, 4)	0.018
*Wall, n (%)*					< 0.001
• Absent	0 (0)	4 (50)	8 (89)	3 (23)	
• Smoth	0 (0)	4 (50)	0 (0)	5 (38)	
• Irregular	3 (100)	0 (0)	1 (11)	5 (38)	
Presence of septa, n (%)	2 (67)	8 (100)	4 (44)	8 (62)	0.086
*Number of septa, n (%)*					0.024
• 0	1 (33)	0 (0)	5 (56)	5 (38)	
• 1	0 (0)	0 (0)	1 (11)	0 (0)	
• 2	0 (0)	0 (0)	0 (0)	2 (15)	
• 3	0 (0)	1 (13)	0 (0)	0 (0)	
• 5	1 (33)	0 (0)	2 (22)	2 (15)	
• > 5	1 (33)	7 (88)	1 (11)	4 (31)	
*Tipe of septa, n (%)*					0.037
• Absent	1 (33)	0 (0)	5 (56)	5 (38)	
• Smoth	0 (0)	2 (25)	3 (33)	1 (7.7)	
• Irregular	2 (67)	6 (75)	1 (11)	7 (54)	
Septa maximum thickness, [mm]	3 (1.5, 3.5)	4.5 (3.75, 5.5)	0 (0, 2)	2 (0, 4)	0.036
Presence of nodules, n (%)	0 (0)	0 (0)	9 (100)	13 (100)	< 0.001
Maximum diameter obtuse angle nodule, [mm]	0 (0, 0)	0 (0, 0)	7 (0, 9)	5 (0, 9)	0.013
Maximum diameter acute angle nodule, [mm]	0 (0, 0)	0 (0, 0)	5 (0, 8)	13 (9, 14)	< 0.001
*Type of nodules, n (%)*					< 0.001
• Absence	3 (100)	7 (88)	0 (0)	0 (0)	
• Obtuse angle nodules alone	0 (0)	1 (13)	0 (0)	0 (0)	
• Acute angle nodules alone	0 (0)	0 (0)	3 (33)	6 (46)	
• Acute and obtuse angles	0 (0)	0 (0)	6 (66)	7 (54)	
*Arterial phase nodule enhancement, n (%)*					< 0.001
No nodule	3 (100)	8 (100)	0 (0)	0 (0)	
• Hypoenhancement in the arterial phase	0 (0)	0 (0)	9 (100)	0 (0)	
• Hyperenhancement in the arterial phase	0 (0)	0 (0)	0 (0)	13 (100)	
Unenhanced attenuation of cystic component, [HU]	16 (14.5, 18.5)	15.5 (13.5, 18.5)	16 (15, 17)	14 (12.8, 19.5)	> 0.9
Arterial phase attenuation of cystic component, [HU]	23 (21.5, 26.5)	20 (18.8, 23)	25 (20, 32)	23 (15, 29)	0.6
Unenhanced attenuation of solid component, [HU]	25 (25, 28.5)	31 (28.8, 33)	29 (27, 38)	30 (29, 35)	0.7
Arterial phase attenuation of solid component, [HU]	56 (55, 90)	125 (121, 133)	90 (77, 125)	232 (225, 240)	< 0.001
Calcifications, n (%)	2 (67)	1 (13)	0 (0)	0 (0)	0.005

*n* number, % = percentage, *HU* (Hounsfield unit), *mm* milimiters, *BC-vs2005* Bosniak Classification version 2005

All CRM-CNP lesions had no thick irregular walls but multiple focal thickened septa (≥ 3). All CRM-PP and CRM-CCP lesions presented acute-angle nodules; however, in CRM-PP group, nodules were smaller (5 mm (IQR, 0–8) vs. 13 mm (9–14); *p* < 0.001). CRM-CCP showed higher arterial phase attenuation of solid nodular component than CRM-PP (232 HU (225–240) vs. 90 HU (77–125); *p* < 0.001). Most ccRCC presented with CRM-CCP pattern (84.61%), and most patients with pRCC presented papillary pattern (88.88%)

Our four pattern proposals were better *p* < 0.001) than BC-v2005 (*p* < 0.001) and BC-v2019 (*p* = 0.022) for differentiating benign and malignant CRM (Table 3). Interreader agreement was almost perfect in assigning the lesional pattern (k = 0.92).

**Table 3:** Performance of classifications to discriminate benignity and malignancy in CRMs

Variable	Benign (*n* = 14; 24.6%)	Malignant (*n* = 43; 75.4%)	*p*-value
*BC v2005, n (%)*			< 0.001
• III	11 (79)	3 (7.0)	
• IV	3 (21)	40 (93)	
*BC v2019, n (%)*			0.022
• III	2 (14)	0 (0)	
• IV	9 (64)	22 (51)	
• Solid	3 (21)	21 (49)	
*Patterns, n (%)*			< 0.001
• Solid	3 (21)	19 (44)	
• 1	3 (21)	0 (0)	
• 2	8 (57)	0 (0)	
• 3	0 (0)	11 (26)	
• 4	0 (0)	13 (30)	

*n* number, % percentage, *BC-vs2005* Bosniak Classification version 2005, *BC-vs2019* Bosniak Classification version 2019

Our four pattern proposals were better *p* < 0.001) than BC-v2005 (*p* < 0.001) and BC-v2019 (*p* = 0.022) for differentiating benign and malignant CRM. Interreader agreement was almost perfect in assigning the lesional pattern (k = 0.92)

Regarding radiopathological correlation, only 7 of the 22 CRMs with a CRM-PP and CRM-CCP had real histological cystic content, with 15 CRM being mucinous, unenhancing papillary, necrotic, or edematous tumors. A review of the pathological findings showed calcifications in two benign hemorrhagic tumors and two pRCC.

## Discussion

The main goals of the new BC-v2019 of CRM are to improve the specificity of malignancy diagnosis of high-risk categories and reduce the proportion of benign lesions that are unnecessarily followed or resected. Neither goal was net in the present study.

In our series, more than half of BC-v2005 Bosniak III/IV CRMs were reclassified as solid tumours by BC-v2019 due to enhancing properties of lesion. No benign Bosniak III CRM by BC-v2005 was downgraded to IIF or lower by BC-v2019. Nine (15.78%) CRM benign (6 MCN and 3 hemorrhagic cysts) considered Bosniak III were upgraded to IV by BC-v2019, due to the presence of enhancing irregular septa or wall and obtuse-angle nodules of more than 3 mm in thickness. In clinical practice, the differentiation of 3 mm to 4 mm measurements is challenging, and this 1 mm change led to an upgrade from BC III to IV with BC-v2019. Indeed, recent studies have reported standardized and specific definitions of morphological features, such as those of BC-v2019, did not improve inter-reader agreement but rather increased the complexity of radiological image interpretation [7, 15, 16, 17]. These studies described that the dispersion among radiologists was largely due to the classification of wall and septa quality, namely in what constituted a nodule versus irregular thickening, and in irregular versus smooth wall and septa qualities, similar to what occurred in the present series [16].

The only statistically significant radiological finding for differentiating benign for malignant CRM in our series was the absence or the presence of acute-angle nodules of any size. Our four patterns proposal differentiating benign and malignant patterns due better than BC 2005 and BC 2019 for benignity and malignancy CRM classifications. Ten from 12 ccRCC CRM presented clear cell pattern with hyperenhancing big nodules in the arterial phase and eight from 9 pRCC CRM showed papillary pattern with hypoenhancing small nodules in the arterial phase, even not perfect pattern differentiation can be useful to differentiate these two RCC subtypes. This nodular size/enhancement differentiation between pCCR and ccRCC subtypes was already profusely described in the literature attending solid renal masses but not in CRM [12, 13].

In accordance with our findings, Pruti et al., and more recently, McGrath et al. proposed to differentiate Bosniak III and IV CRM with irregular septa and walls from those which have clearly defined nodules [6, 8]. In the present series the first, with thick septa and/or walls, corresponds to benign CRM-IP and CRM-CNP, while the second with acute angle nodules corresponds to malignant CRM-PP and CRM-CCP.

The American and Canadian Urological Associations and the European Urological Association actualized guidelines suggest active surveillance for any Bosniak III and IV masses of less than 4 cm [18, 19]. Follow-up of CRM can be safely performed by CEUS, CT and MR and AS can be discontinued if increased solid tissue in septa, walls and nodularity is found, even if the diameter of the CRM remains stable. [10, 20, 21]. This would allow a reduction in surgical resections of benign BC-v2019 Bosniak III/IV CRMs.

The present study has several limitations. First, it is a single-center retrospective study and only includes surgically or biopsy proven CRM, leading to selection and verification biases with a high proportion of malignant masses. Second, MRI findings were not included. Third, the sample was small and so it is difficult to obtain solid conclusions from this, specially for the description of inflammatory lesions, although previous series of surgically managed CRMs with pathological correlation also included similar samples sizes. [22, 23]. There is a large number of BC-v2005 that were considered solid by BC-v2019, with a possible bias of what we consider cystic in our center. Lastly, in the case of disagreement between the two readers in the interpretation of the radiological findings, a third reader decided with a possible bias in favor of this third radiologist.

In conclusion, the proposed four radiological patterns improved performance than BC-v2019 in classifying benign lesions (CRM-IP and CRM-CNP) and malignant ones (CRM-PP and CRM-CCP), with wall and septa thickening and acute-angle nodules as relevant biomarkers.

## Electronic supplementary material

Below is the link to the electronic supplementary material.

Supplementary Material 1.

## Data Availability

No datasets were generated or analysed during the current study.

## Abbreviations

AS: Active surveillance
BC: Bosniak classification
ccRCC: Clear cell renal cell carcinoma
CEUS: Contrast-enhanced ultrasound
Cr: Creatinine
CRM: Cystic renal mass
CRM-CCP: Cystic renal mass clear cell pattern
CRM-CNP: Cystic renal mass cystic nephroma pattern
CRM-IP: Cystic renal mass inflammatory pattern
CRM-PP: Cystic renal mass papillary pattern
CT: Computed tomography
GFR: Glomerular filtration rate
HU: Hounsfield units
MCN: Multilocular cystic nephroma
MRI: Magnetic resonance imaging
pRCC: Papillary renal cell carcinoma
v2005: Version 2005
v2019: Version 2019

## References

[1] Bosniak M. A. (1986). The current radiological approach to renal cysts. Radiology, 158(1), 1–10. 10.1148/radiology.158.1.3510019 3510019 10.1148/radiology.158.1.3510019

[2] Israel, G. M., & Bosniak, M. A. (2005). An update of the Bosniak renal cyst classification system. Urology, 66(3), 484–488. 10.1016/j.urology.2005.04.003 16140062 10.1016/j.urology.2005.04.003

[3] Silverman, S. G., Pedrosa, I., Ellis, J. H., Hindman, N. M., Schieda, N., Smith, A. D., Remer, E. M., Shinagare, A. B., Curci, N. E., Raman, S. S., Wells, S. A., Kaffenberger, S. D., Wang, Z. J., Chandarana, H., & Davenport, M. S. (2019). Bosniak Classification of Cystic Renal Masses, Version 2019: An Update Proposal and Needs Assessment. Radiology, 292(2), 475–488. 10.1148/radiol.2019182646 31210616 10.1148/radiol.2019182646 PMC6677285

[4] Park, M. Y., Park, K. J., Kim, M. H., & Kim, J. K. (2021). Bosniak Classification of Cystic Renal Masses Version 2019: Comparison with Version 2005 for Class Distribution, Diagnostic Performance, and Interreader Agreement Using CT and MRI. *AJR. American journal of roentgenology*, *217*(6), 1367–1376. 10.2214/AJR.21.25796 10.2214/AJR.21.25796 34076460

[5] Zhang, Q., Dai, X., & Li, W. (2022). Diagnostic performance of the Bosniak classification, version 2019 for cystic renal masses: A systematic review and meta-analysis. *Frontiers in oncology*, *12*, 931592. 10.3389/fonc.2022.931592 36330503 10.3389/fonc.2022.931592 PMC9623069

[6] McGrath, T. A., Bai, X., Kamaya, A., Park, K. J., Park, M. Y., Tse, J. R., Wang, H., McInnes, M. D. F., & Schieda, N. (2023). Proportion of malignancy in Bosniak classification of cystic renal masses version 2019 (v2019) classes: systematic review and meta-analysis. European radiology, 33(2), 1307–1317. 10.1007/s00330-022-09102-w 35999371 10.1007/s00330-022-09102-w

[7] Dana, J., Gauvin, S., Zhang, M., Lotero, J., Cassim, C., Artho, G., Bhatnagar, S. R., Tanguay, S., & Reinhold, C. (2023). CT-based Bosniak classification of cystic renal lesions: is version 2019 an improvement on version 2005?. European radiology, 33(2), 1297–1306. 10.1007/s00330-022-09082-x 36048207 10.1007/s00330-022-09082-x

[8] Pruthi, D. K., Liu, Q., Kirkpatrick, I. D. C., Gelfond, J., & Drachenberg, D. E. (2019). Long-Term Surveillance of Complex Cystic Renal Masses and Heterogeneity of Bosniak 3 Lesions. The Journal of urology, 10.1097/JU.0000000000000144. Advance online publication. 10.1097/JU.0000000000000144 30747876 10.1097/JU.0000000000000144

[9] Chandrasekar, T., Ahmad, A. E., Fadaak, K., Jhaveri, K., Bhatt, J. R., Jewett, M. A. S., & Finelli, A. (2018). Natural History of Complex Renal Cysts: Clinical Evidence Supporting Active Surveillance. The Journal of urology, 199(3), 633–640. 10.1016/j.juro.2017.09.078 28941915 10.1016/j.juro.2017.09.078

[10] Sebastià, C., Corominas, D., Musquera, M., Paño, B., Ajami, T., & Nicolau, C. (2020). Active surveillance of small renal masses. Insights into imaging, 11(1), 63. 10.1186/s13244-020-00853-y 32372194 10.1186/s13244-020-00853-y PMC7200970

[11] Wood, C. G., 3rd, Stromberg, L. J., 3rd, Harmath, C. B., Horowitz, J. M., Feng, C., Hammond, N. A., Casalino, D. D., Goodhartz, L. A., Miller, F. H., & Nikolaidis, P. (2015). CT and MR imaging for evaluation of cystic renal lesions and diseases. *Radiographics: a review publication of the Radiological Society of North America, Inc*, *35*(1), 125–141. 10.1148/rg.351130016 25590393 10.1148/rg.351130016

[12] Shen, J., & Zou, Y. (2024). Diagnostic value of contrast-enhanced CT in clear cell renal cell carcinoma: a systematic review and meta-analysis. *BMC urology*, *24*(1), 189. 10.1186/s12894-024-01574-w 39218886 10.1186/s12894-024-01574-w PMC11368016

[13] Dilauro, M., Quon, M., McInnes, M. D., Vakili, M., Chung, A., Flood, T. A., & Schieda, N. (2016). Comparison of Contrast-Enhanced Multiphase Renal Protocol CT Versus MRI for Diagnosis of Papillary Renal Cell Carcinoma. *AJR. American journal of roentgenology*, *206*(2), 319–325. 10.2214/AJR.15.14932 26797358 10.2214/AJR.15.14932

[14] Pitra, T., Pivovarcikova, K., Alaghehbandan, R., Bartos Vesela, A., Tupy, R., Hora, M., & Hes, O. (2022). A Comprehensive Commentary on the Multilocular Cystic Renal Neoplasm of Low Malignant Potential: A Urologist’s Perspective. *Cancers*, *14*(3), 831. 10.3390/cancers14030831 35159098 10.3390/cancers14030831 PMC8834316

[15] Elbaset, M. A., Ashour, R., Elgamal, M., Elbatta, A., Ghobrial, F. K., Abouelkheir, R. T., Mosbah, A., & Osman, Y. (2023). The efficacy of the new Bosniak classification v.2019 in benign lesions prediction within the higher Bosniak cysts classes. *Urologic oncology*, *41*(10), 434.e1–434.e7. 10.1016/j.urolonc.2023.06.007 37574368 10.1016/j.urolonc.2023.06.007

[16] Shampain, K. L., Shankar, P. R., Troost, J. P., Galantowicz, M. L., Pampati, R. A., Schoenheit, T. R., Shlensky, D. A., Barkmeier, D., Curci, N. E., Kaza, R. K., Khalatbari, S., & Davenport, M. S. (2022). Interrater Agreement of Bosniak Classification Version 2019 and Version 2005 for Cystic Renal Masses at CT and MRI. Radiology, 302(2), 357–366. 10.1148/radiol.2021210853 34726535 10.1148/radiol.2021210853 PMC8805658

[17] Bai, X., Sun, S. M., Xu, W., Kang, H. H., Li, L., Jin, Y. Q., Gong, Q. G., Liang, G. C., Liu, H. Y., Liu, L. L., Chen, S. L., Wang, Q. R., Wu, P., Guo, A. T., Huang, Q. B., Zhang, X. J., Ye, H. Y., & Wang, H. Y. (2020). MRI-based Bosniak Classification of Cystic Renal Masses, Version 2019: Interobserver Agreement, Impact of Readers’ Experience, and Diagnostic Performance. Radiology, 297(3), 597–605. 10.1148/radiol.2020200478 32960726 10.1148/radiol.2020200478

[18] Richard, P. O., Violette, P. D., Bhindi, B., Breau, R. H., Gratton, M., Jewett, M. A. S., Kapoor, A., Pouliot, F., Leveridge, M., So, A. I., Whelan, T. F., Rendon, R. A., Tanguay, S., & Finelli, A. (2023). 2023 UPDATE - Canadian Urological Association guideline: Management of cystic renal lesions Prior to original publication (March 2017), this guideline underwent review by the CUA Guidelines Committee, CUA members at large, and the CUA Executive Board. The 2023 updates were app roved by the CUA Guidelines Committee and CUA Executive Board. *Canadian Urological Association journal = Journal de l’Association des urologues du Canada*, *17*(6), 162–174. 10.5489/cuaj.8389 10.5489/cuaj.8389 PMC10263289 37310905

[19] https://uroweb.org/guidelines/renal-cell-carcinoma

[20] Lee, R. A., Uzzo, R. G., Anaokar, J., Thomas, A., Wei, S., Ristau, B. T., McIntosh,A., Lee, M., Chen, D. Y. T., Greenberg, R. E., Viterbo, R., Smaldone, M. C., Correa,A., Schober, J., Ginsburg, K., Bukavina, L., Magee, D., Uzzo, N., Parkansky, P., Ruth,K.,… Kutikov, A. (2023). Pathological and Clinical Outcomes in a Large Surveillance and Intervention Cohort of Radiographically Cystic Renal Masses. The Journal of urology,209(4), 686–693. 10.1097/JU.0000000000003158 10.1097/JU.0000000000003158 36630588

[21] Möller, K., Jenssen, C., Correas, J. M., Safai Zadeh, E., Bertolotto, M., Ignee, A., Dong, Y., Cantisani, V., & Dietrich, C. F. (2023). CEUS Bosniak Classification-Time for Differentiation and Change in Renal Cyst Surveillance. Cancers, 15(19), 4709. 10.3390/cancers15194709 37835403 10.3390/cancers15194709 PMC10571952

[22] Lucocq, J., Morgan, L., Rathod, K., Szewczyk-Bieda, M., & Nabi, G. (2023). Validation of the updated Bosniak classification (2019) in pathologically confirmed CT-categorised cysts. Scottish medical journal, 369330231221235. Advance online publication. 10.1177/00369330231221235 10.1177/00369330231221235 38111318

[23] Perri, D., Mazzoleni, F., Pacchetti, A., Rossini, M., Morini, E., Berti, L., Buizza, C., Besana, U., & Bozzini, G. (2023). Pathological report and prognostic meaning of Bosniak IV cysts: results from a contemporary cohort. Central European journal of urology, 76(3), 186–189. 10.5173/ceju.2023.083R 38045787 10.5173/ceju.2023.083R PMC10690387

