# Supplementary material for: Defining new radiological patterns to improve classification of Bosniak III and IV cystic renal masses
Source: Abdom Radiol (NY). 2025 Jul 2;51(2):854–64. doi: 10.1007/s00261-025-05092-7 (PMC12929308; doi:10.1007/s00261-025-05092-7)
Supplement: Supplementary file 1 — Supplementary Material 1. [file 261_2025_5092_MOESM1_ESM.pptx]

## Slide 1
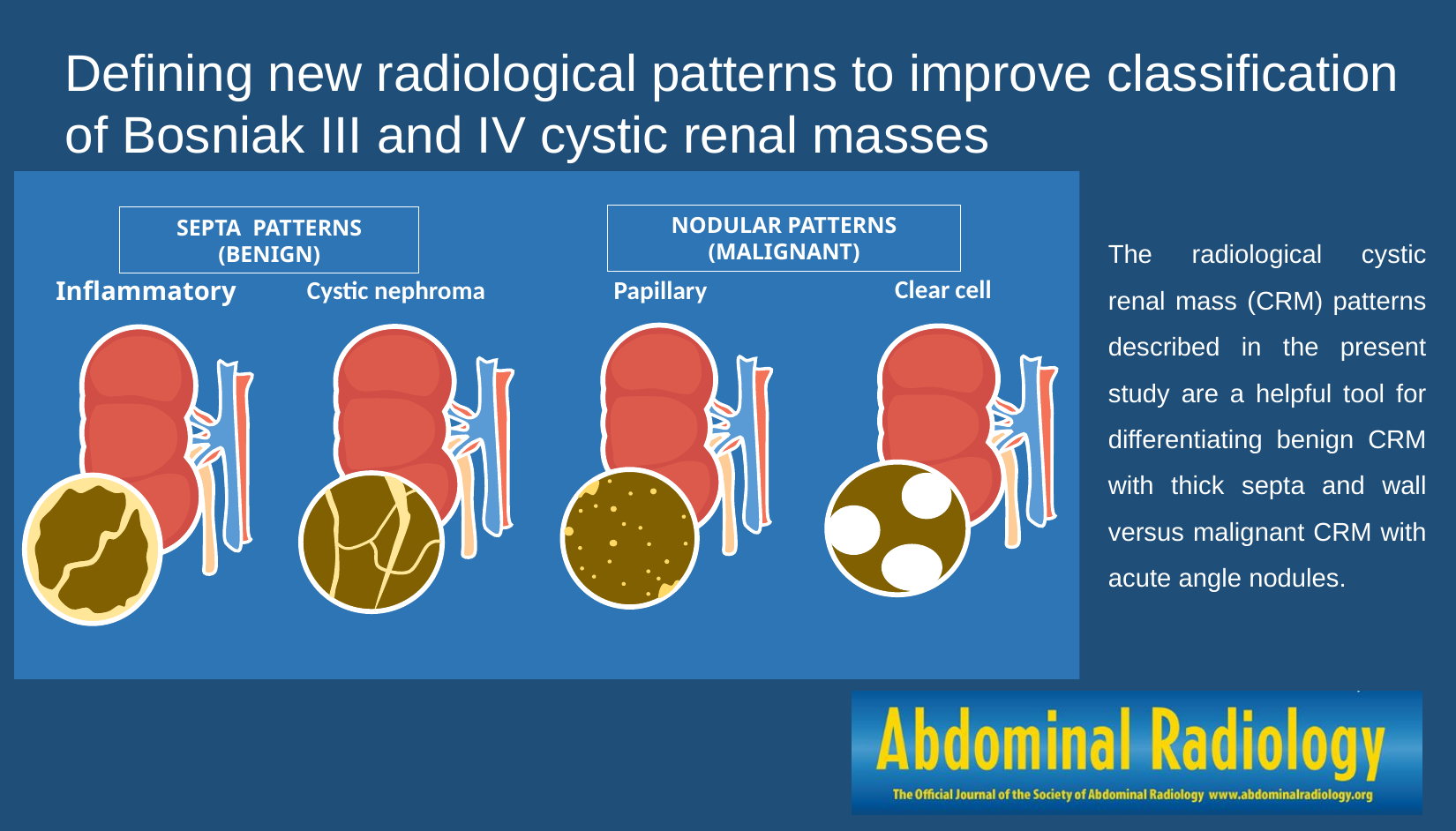

Defining new radiological patterns to improve classification of Bosniak III and IV cystic renal masses
The radiological cystic renal mass (CRM) patterns described in the present study are a helpful tool for differentiating benign CRM with thick septa and wall versus malignant CRM with acute angle nodules.
NODULAR PATTERNS (MALIGNANT)
SEPTA PATTERNS (BENIGN)
Clear cell
Papillary
Cystic nephroma
Inflammatory
